# A qualitative analysis of stakeholder experiences with Registered Reports Funding Partnerships

**DOI:** 10.12688/wellcomeopenres.17029.1

**Published:** 2021-09-14

**Authors:** Katie Drax, Robbie Clark, Christopher D. Chambers, Marcus Munafò, Jacqueline Thompson

**Affiliations:** 1 School of Psychological Science, University of Bristol, Bristol, BS8 1TU, UK; 2 MRC Integrative Epidemiology Unit, University of Bristol, Bristol, BS8 2PR, UK; 3 School of Psychology, Cardiff University, Cardiff, CF10 3AT, UK

**Keywords:** Registered Reports, research funding, partnerships, research publishing, qualitative, thematic analysis

## Abstract

**Background:** Registered Reports (RRs) could be a way to increase the quality of scientific research and literature, such as by reducing publication bias and increasing the rigour of study designs. These potential benefits have led to Registered Report funding partnerships (RRFPs or partnerships for short) between research funders and academic journals who collaborate to encourage researchers to publish RRs. In this study we investigated the research question: “What are the experiences of the stakeholders (authors, reviewers, journal editors, funders) in the various partnership models?”. Our companion paper addresses a related, but separate, research question.

**Methods:** We conducted a thematic analysis of 32 semi-structured interviews with stakeholders (funders, editors, authors, reviewers, matchmakers) from six partnerships.

**Results:** Interviewees had highly variable perceptions and experiences, reflecting the complex and nuanced impacts of partnerships. We identified 6 themes: “Importance of communication with authors and reviewers”, “Influence on study design”, “Appropriateness of partners”, “Potential to reduce publication bias”, “Impact on reviewer workload”, and “Insufficient evidence”.

**Conclusions:** This was the first investigation into these novel initiatives. We hope that our findings can benefit and shape current and future partnerships.

## Introduction

Registered Reports (RRs) are a research report format. Started at *Cortex* in 2013, RRs undergo two rounds of peer review, once before data collection or analysis (Stage 1) and once after (Stage 2). For Stage 1, authors submit a study protocol containing the Introduction and Methods sections, which reviewers then assess. Protocols that pass Stage 1 are granted in-principle acceptance (IPA), meaning they cannot be rejected based on the main results they report in Stage 2. At Stage 2 peer review, authors submit the completed manuscript containing the Results and Discussion sections and reviewers check authors adhered to protocol from Stage 1. These two components, pre-study review and IPA, are what define an RR ^1^.

Authors of articles discussing RRs cite benefits of IPA and pre-study peer review. These benefits are mostly theoretical since little empirical evidence exists about RRs, although some early work is available ^2– 6^. One frequently cited benefit is the possibility for IPA to reduce publication bias for certain types of results and to disincentivise questionable, and invalid, research practices used to obtain these more favourable results ^7– 10^. Another proposed benefit is how pre-study peer review may improve study designs ^11– 13^. Contrastingly, a frequent concern about RRs is that they may take more time and effort ^14– 16^.

As of June 2021, 294 journals offer RRs ^1^ and some academic journals and research funders are joining together to encourage researchers to publish in the RR format. We refer to these collaborations between funders and journals that offer Registered Reports as Registered Report funding partnerships (RRFPs or partnerships for short). Early proposals of partnerships came from 17 and 18. The difference between submitting to a RR journal or to a partnership is the role of the funder. In the conventional research process, funders are rarely involved in the publication of their grantees’ research. Conversely, funders in partnerships encourage grantees to publish in a specific journal and communicate directly with that journal. This involvement can vary from light touch (e.g., suggesting that grantees publish their funded research as an RR) to greater involvement (e.g., requiring that grantees obtain IPA from a journal before receiving funding, or sharing grant reviews with the journal).

In practice, the design and logistics of partnerships vary greatly; there is no single agreed format. The existing partnerships all involve one funder and one RR journal, collaborating to streamline the research process from funding application to publication.

### Rationale for study

The number of journals offering RRs is increasing and at least five funder-RR journal partnerships already exist. This uptake demonstrates that many journals and several funders believe the potential benefits of RRs outweigh their potential downsides, but the true effect of RRs on the funding and publishing processes remains unknown. A randomised control trial (RCT) comparing typical funding processes against RR partnerships would provide convincing evidence to assess the impacts of RR partnerships. However, given our limited understanding of how RR funding partnerships work in practice it is difficult to know how an RCT should be designed. We therefore conducted a qualitative feasibility study to inform a pilot RCT that will assess the impacts of partnerships. Here we use the definition of feasibility studies set out by Eldridge and colleagues ^19^. Our study investigated the two research questions related to design and delivery of the intervention and future RCT. 20’s framework, describing what aspects of RCTs qualitative research can improve, informed the formulation of both research questions. This paper deals with the first research question: “What are the experiences of the stakeholders (authors, reviewers, journal editors, funders) in the various partnership models?”. Our companion paper, led by RC, handled our second research question that aimed to investigate various factors relating to the feasibility of a partnership RCT ^72^. Note that this was a deviation from our protocol, in which we aimed to investigate the question: “What outcome measures of an RCT will be valid, reliable, feasible, acceptable and yield high completion rates?”. We preregistered our protocol on the OSF after conducting four interviews but before we transcribed or analysed any interviews ^71^.

## Methods

### Study design

To answer our research question regarding the experiences of stakeholders we conducted a thematic analysis of semi-structured interviews about participants’ experiences and opinions of partnerships. Semi-structured interviews were chosen over other interview formats to ensure that essential questions were answered by participants while allowing follow-up questions to be asked if important topics emerged. They were also preferable to focus groups as we expected them to be easier to organise for a target population that is small and geographically dispersed.

### Recruitment

Using Internet searches, personal communications, and the Center for Open Science hub for RRs ^28^, we identified seven potential partnerships, six active and one in development.

After discussion with two individuals involved in the PCF-PeerJ scheme, we learned that authors conducted RRs because of personal choice, not because the funders encouraged them to. Consequently, we decided that PCF and PeerJ did not meet our criteria of a partnership. This left a final sample of six partnerships, five active and one in development. See Supplementary Material for a detailed description of the six partnerships timelines and processes.

We used a convenience and snowball sampling method for recruitment ^29^. Anyone who was over 18 and had experience as a reviewer, author, editor, funder, or other role in a partnership could participate. No compensation was given for participation. First, we identified prospective participants using publicly available information and from our existing relationships with personnel from the journals and funders involved in partnerships. We then emailed them an invitation to be interviewed. We also asked editors and funders to recommend authors and reviewers who may qualify for participation. Where possible we contacted authors and reviewers directly, otherwise the funder or journal contacted them for us. We followed up on non-responses, waiting at least a week, sending a maximum of three emails. All the funders and editors we contacted agreed to participate. Of the 39 authors and reviewers contacted, 14 agreed to participate, 19 never replied, 2 stopped following up, 2 asked to follow up much later, and 2 declined.

### Participants

We conducted 32 semi-structured interviews with people from five stakeholder groups. These were: authors of partnership submissions (“authors”), personnel at partner funders (“funders”), editors at partner journals (“editors”), reviewers of partnership submissions (“reviewers”), and personnel who help to set-up or run a partnership but are not affiliated with its funder or journal (“matchmakers”). We retrospectively defined the “matchmaker” group after the interviews.

We aimed to recruit at least one person from each partnership for each of their relevant stakeholder groups to help us understand the full range of experiences in each of the partnerships. We aimed to achieve the equal distribution across the cells shown in Table 1.

**Table 1.** Sample.

Partnership	Author	Editor	Funder	Reviewer	Matchmaker
APLS-PLS	1	2	2	0	NA
CRUK-N&TR	1	1	3	3	NA
CTF-PLOS	2	4	1	2	NA
Flu Lab-PLOS-COS	NA	3	1	NA	2
Pfizer-N&TR	4	1	1	1	NA
PLOS Bio-CHDI	NA	3	1	NA	NA

*APLS-PLS is the partnership between The Association for Politics & The Life Sciences (APLS) and Politics and the Life Sciences journal (PLS) ^25^.* *CRUK-N&TR is the partnership between Cancer Research UK’s Tobacco Advisory Group (TAG) and Nicotine & Tobacco Research journal ^17^*. *CTF-PLOS is the partnership between Children’s Tumor Foundation (CTF) and PLOS ONE ^24^*. *Flu Lab-PLOS-COS is the partnership between The Flu Lab, PLOS One and the Center for Open Science ( https://cos.io/our-services/research/flulab/)* *Pfizer-N&TR is the partnership between Global Research Awards for Nicotine Dependence and Nicotine & Tobacco Research journal ^23^*. *PLOS Bio-CHDI is the partnership between PLOS Biology and the CHDI Foundation ^27^ which is not yet open for submissions*.

Table 1 shows the actual distribution we achieved. More information about our interviewees’ characteristics is available at https://doi.org/10.5523/bris.1m38wyz9gvzo52i2kpecr8w6kb upon application. As can be seen, we covered most cells of Table 1 with at least one interviewee. We had higher samples in some cells as we did not wish to turn away additional willing interviewees. This also means that we did not recruit people on the basis of data saturation. We interviewed members of all relevant stakeholder groups for CRUK-N&TR, CTF-PLOS, and Pfizer-N&TR. We interviewed no reviewers from the APLS-PLS partnership. This was the only partnership we failed to interview representatives from all applicable stakeholder groups. Cells in Table 1 add up to 39 because four editors were editors for multiple partnerships and two editors were also funders. The two matchmakers were representatives from the Center for Open Science (COS); COS facilitated the partnership between The Flu Lab and PLOS One. For more information about the partnerships and their processes please see Appendix A of our protocol ^71^.

### Materials

The study required interview guides. Given that editors, funders, reviewers, and authors would be asked different questions we designed four interview guides, one for each group. For the interviews with matchmakers, we used the funders’ interview guide. The four final versions of each guide are available on the OSF project ^30^. Briefly, they all included questions on the partnership’s strengths, weaknesses, areas for improvement, impact on research quality, efficiency of research process, and the interviewees reasons for getting involved in the scheme. We additionally asked funders and editors about their experience setting up, designing, and implementing the partnership.

### Pilot

JT, KD and RC piloted the interview guides on each other to confirm the appropriateness and ordering of the questions, practicing interview technique while doing so. We conducted further piloting with, and feedback obtained from, people with expertise in both qualitative research and/or Registered Reports. In total we spoke to 14 people, between 12 December 2019 and 7 February 2020. This helped to further refine our technique. We also adapted the interview guides. While the subject and sequence of most questions in the guides remained similar we made changes mainly to questions’ wording, tone, and potential follow-up probes. We also added reminders to the guides to help us with the interviews, such as reminders to do a sound check.

### Procedure

We obtained ethics approval for this study from the School of Psychological Science Research Ethics Committee at the University of Bristol (Approval Code: **06022098163**). Selected participants were invited by email. When an individual agreed to participate, we emailed them the information sheet and online consent form. The interviewee confirmed a convenient time for the interview. Interviewees signed the consent form before beginning the interview. Given the ongoing coronavirus disease 2019 (COVID-19) pandemic, all interviews were remote, using the video conferencing software Bluejeans and Webex. JT, KD, and RC were present at almost all interviews and each led roughly a third of the interviews. They each introduced themselves to the interviewee at the start of the interview. Anyone not leading the interview turned off their video and microphone and the interviewer invited them to turn it back on once the interview was over. RC recorded all interviews on a handheld audio recorder positioned close to his computer speaker. RC, KD, and JT kept field notes during the interviews. All interviews occurred between 19 March 2020 and 4 August 2020. They lasted between 23 and 97 minutes. The mean duration was 60 minutes. See Table 2 for a summary of KD, JT, and RC’s previous experience of qualitative research.

**Table 2.** Interviewer characteristics.

Interviewer	Credentials	Occupation	Gender	Experience & training	Participant knowledge of interviewer	Interviewer’s views about the research topic
KD	BSc	PhD student	Female	One semester-long module at undergraduate	KD is well-acquainted with one participant; Active Twitter user which participants may access	Believes RRs are likely best practice for confirmatory research
JT	PhD	Post doctorate researcher	Female	Taught undergraduate module on thematic analysis; one-day course on qualitative interviewing; 30+ phenomenology interviews for cognitive study	Email contact and in- person meeting with potential participants from funders and editors; well-acquainted with one participant; Active Twitter user which participants may access	Written one RR; given workshops advocating for RRs and believes they are likely best practice for confirmatory research
RC	MSc	Research associate	Male	Has studied the fundamentals of qualitative research during Research Methods module in MSc. Experience leading around 12 interviews and focus groups in academic settings.	Well-acquainted with one participant; Active Twitter user which participants may access	Believes RRs could provide a useful means of overcoming publication bias and encouraging transparent research

Bristol Transcription Services transcribed 12 interviews. RC listened to the audio recordings of these transcripts, corrected any inaccuracies, and ensured the notation was consistent with the other transcripts. RC transcribed the other 20 interviews. We did not transcribe one interview because they were involved in the PCF-PeerJ scheme which, as explained in the Introduction, we decided subsequently did not meet our definition of a partnership. To ensure data quality, KD listened to the audio recordings of more than 4 (10%) of the transcripts and compared them to their accompanying transcript produced by RC. KD discussed any inaccuracies with RC and corrected them if necessary. KD analysed the interviews using NVIVO 12 (released in March 2020), with feedback from MM, JT, and RC. KD used ^31^ to convert NVIVO nodes into a codebook and ^32^ to write the manuscript. All R packages she used are cited in the References and code is available as Extended data ^71^.

### Positionality statement

We hoped our interviews would provide useful feedback to organisations currently involved in partnerships. As such, we took an almost business-like approach to evaluating partnerships’ strengths, weaknesses, potential improvements, and so on. In our approach to the interviews we took people “at their word” while being mindful of some factors that could influence their accounts and our interpretation of them. For example, we anticipated that funders and editors might present their experiences positively because they have a stake in the success of the initiative. Likewise, we all believe that RRs will benefit some areas of research (see Table 2) so we may have a bias towards favourable interpretations of interviewees’ accounts. In our interview guide we explicitly aimed to ask people about topics they neglected. For example, if we believed participants focused on the positives, we made sure to ask follow-up questions about any negative aspects.

KD took a similar approach to the thematic analysis. She assumed that interviewees’ accounts accurately reflected their experiences. She appreciated their accounts and experiences would be affected by the contexts they occurred in. This was especially clear interviewees’ understandings of their relevant partnership conflicted with how we knew the partnership process worked, when reviewers did not realise the manuscripts were a RR, or when interviewees could not remember details of their experience. Beyond the factors of memory and understanding affecting people’s accounts, KD largely discounted the influence of social identities, power structures, and their intersections on interviewees’ accounts and experiences.

### Analysis plan

KD analysed the interview transcripts after we finished the interviews. She analysed them in no particular order, using thematic analysis following the step-by-step guide from Braun and colleagues ^21^. KD’s technique changed over time and according to the transcript being analysed so we cannot describe it as a linear, step-by-step process. Instead, the analysis process was iterative and moved between Braun and colleagues’ steps throughout.

1.      Familiarising yourself with your data.

KD skim-read transcripts or listened to the audio recordings of interviews.

2.      Generating initial codes.

She labelled the data with keywords or short phrases that described them or interpret their meaning. Almost all codes were data-driven because the lack of existing theory or understanding of the effects of partnerships meant we did not wish to not code data with any pre-existing themes in mind. We define a theme as a “pattern of shared meaning, organised around a core concept” or idea/observation ^33^. However, the field notes reveal we anticipated some patterns in the interviews before the analysis began. For example, the notes frequently refer to what became the “Importance of communication with authors and reviewers” and Insufficient evidence” themes. As such, the field notes informed some codes and themes.

3.      Searching for themes.

KD found coded extracts and grouped them into themes and sub-themes. She visualised this by creating a .csv file containing all the codes and their corresponding coded text.

5.      Reviewing themes.

KD reviewed and refined codes, sub-themes and themes using two techniques.

a)      Check codes and themes fit each other.

She checked codes and themes were distinct and non-repetitive, and recoded or combined those that were not. She read through the coded extracts to check the themes and codes matched their supporting data. If they did, she recoded or rethemed them. She focused on the most frequent and widespread codes since we were interested in how the partnerships affected all stakeholders. Infrequent themes or codes were largely ignored during the write up and she tried to incorporate them once she had written the main body of the analysis. She discarded themes and codes she could not incorporate.

b)      Check codes and themes fit entire dataset.

KD attempted to reflect on her analysis to check that it accurately reflected the entire dataset.

To help guide her in these two techniques, KD used the following questions to check her themes did have a core concept.

•      Does the theme describe a pattern of meaning across the dataset?

•      Are these codes organised around a core concept? If no, is it a “domain summary” with nothing tying the codes together?

•      Does the map of codes and themes sufficiently match the entire dataset?

5.      Defining and naming themes.

KD gave short and descriptive names to themes. KD wrote as she collated these themes to analyse what insight each theme contains, how the themes relate to each other and what insights the themes give into the dataset. KD repeated steps 1–5 while she wrote up her analysis of the themes, codes, and data to answer the research questions. This helped to further refine themes and to incorporate any infrequent or underdeveloped sub-themes and codes.

## Results

People representing our four stakeholder groups expressed divergent opinions on the impacts, potential improvements, and scalability of their respective partnerships. Our research question asked, “What are the experiences of the stakeholders (authors, reviewers, journal editors, funders) in the various partnership models?”. In response to this question, KD constructed six themes through her analysis: “Importance of communication with authors and reviewers”, “Influence on study design”, “Appropriateness of partners”, “Potential to reduce publication bias”, “Impact on reviewer workload”, and “Insufficient evidence”.

### Importance of communication with authors and reviewers

Interviewees underlined the importance of clear communication between all stakeholders. KD built this theme from the numerous comments indicating: the need for better or more communication about the partnership, misunderstandings about how the partnership worked, negative consequences from misunderstandings, and the need for stakeholders to pay more attention to the information they receive. KD believed this to be the most common theme she constructed because of the frequency and richness of evidence across all interviews.

Four funders, all editors, and all matchmakers discussed their efforts to ensure potential or existing stakeholders understood the process and their requirements. These efforts included: targeting their communities with an advertising or educational campaign, choosing to work with people already familiar with RR publication or the partnership processes, and directly passing information to the handling editors, authors, and reviewers. The purposes of these efforts at communication tended to differ by stakeholder type, however. The funders and matchmakers focused on the need for potential authors to understand and be aware of their partnership, so that authors would want to submit. In contrast, editors were most vocal about the need for reviewers and handling editors to understand the process so that it worked smoothly. Every editor spoke about the benefits of all stakeholders being familiar with the RR format, the problems when people were unfamiliar, and the efforts they made to ensure people understood the RR format. E1, E2, E3, E6, E7 and E8 all developed resources to explain RRs to their reviewers or editorial board. E7, and E8 also invited reviewers and editorial board members who they knew were already familiar with RRs. To ensure someone who understood the process handled the submissions, E5 handled all submissions themselves. In summary, the educational efforts interviewees described cost them time and effort but appear to have been at least somewhat unsuccessful in effectively informing, or even reaching, the target audience.

While editors, funders, and matchmakers appeared to understand the partnership and RR process, authors and reviewers often did not. Many authors and reviewers were unsure or misunderstood some aspect of the partnership, RR process, author requirements or reviewer requirements. For example, F1 and E5 spoke with authors who mistakenly thought the Stage 1 manuscript would be its own publication. This was corroborated by A1 and A4 who both made this mistake and A4 withdrew their manuscript from the journal as a result. Another misunderstanding was around deviations. Co-authors A5 and A6 were unsure about the freedom to deviate from their in-principally accepted Stage 1 manuscript, which led to stress or concern about the Stage 2 being rejected or more scrutinised by reviewers. Had A5 and A6 declared their deviations to the journal editors prior to submitting their Stage 2 manuscript they may have felt more confident that the Stage 2 review would go smoothly. Misunderstandings by authors and reviewers can make the partnership process more labourious. R1 and M1 both handled submissions from authors who misunderstood the requirements, leading to M1 rejecting some submissions and R1 providing authors with detailed comments for improving their submission. E2 and E6 had to work to resolve issues when a reviewer did not review according to the reviewer requirements. R2 and R3 corroborated this because they did not realise that the manuscripts that they reviewed were RRs or part of a partnership. The confusion created additional work for E2 who stated:

*E2: For the Flulab submission it also takes more time because again, we did face this situation where one of the reviewers had not, in our opinion, evaluated the paper per the registered report framework. So it means that we had to go back and intervene, provide clarification, involve a different editor to make sure that the framework is correct.*

One reason for authors’ and reviewers’ ignorance and misunderstandings is that they did not always read the information they received about the partnerships or RRs. A4 and their team did not fully read the emails explaining the scheme, and therefore mistakenly believed that opting-in would increases chances of application success. When they did read about the process, they realised the Stage 1 manuscript would not be a separate publication, and had to withdraw. E5 speculated that reviewers did not read the invitation email, and R2 corroborated this suspicion by reporting that they indeed had not. R2 believed that not reading review invitation emails was part of a larger behaviour trend, suggesting that some portion of reviewers might be unaware that the paper they reviewed was an RR or part of a partnership.

*R2: I just saw the ‘invitation to review’, read the title, checked that it was within my expertise, probably went down to read the abstract, that’s my usual process, and then clicked on the Agree/Disagree, whatever. Partly because usually the information below in the email is just sort of standard bumpf and, again, because I’ve reviewed for NTR before, I didn’t really pick up that this was anything different. So in answer to your question, I think good old-fashioned bold [laughs] or double asterisks saying, this is a registered report which means – that bit just kind of blurs into the text of the email which, as I said, because we know it’s standard, we tend to ignore it.*

*Interviewer: (49:07) Yeah.*

*R2: (49:08) It’s probably that most other people are a bit more conscientious than me and that they’re better at reading these things but I probably do represent at least some academics that probably habituate to a process, particularly when you do peer reviews. And it’s something, as I said, that you have limited time to do so you just want to get it over and done with, if you like [laughs]. I don’t mean that in a negative way, but it is something that feels like a bit of a chore sometimes, but an important chore.*

As R2 suggests, scientific communities may generally disregard journal and editorial guidelines. If R2 is correct, such behaviour may cause difficulties for partnerships, as researchers who fail to read their emails may be uninformed or misinformed on key details. However, interviewees’ comments about the communications they received about the partnerships suggests the failure in communications does not lie solely with recipients not reading their emails.

Authors, reviewers, and editors criticised existing communications or requested improvements suggesting that partner organisations did not always have clear, sufficient, attention-grabbing, and engaging communication. A1, A4, A7, R1, R2, R3, R5, F1, E4, E5, and E6 all expressed a need for more or better communication to resolve their own or others’ incorrect and incomplete understandings. R5 demonstrated that the emails could fail to fully inform reviewers about the partnership process.

*Interviewer: Thinking about the logistics of the process, has anything worked well or poorly?*

*R5: (37:08) No, I think, yes actually I do remember I think corresponding with [E5] initially, asking how I should approach this. And I think I had some follow-up questions before submitting my review as to what is the role of this review and how should I position myself; these were the questions I had. And I felt the initial email didn’t necessarily include all the information to really help me as a reviewer position myself and go through the process. So maybe if there was an FAQ somewhere, I mean there are clear rules actually of what is happening*

This is not to say that asking further questions for clarification means that written material is insufficient or poorly designed. Both A5 and A8 asked further questions to the editors. Unlike R5, however, both felt the written instruction they received was sufficient. As noted above A5 could have benefited from clearer guidance on deviations, but A8 demonstrated a complete understanding of the process. We asked them specifically about the communications they received from the editor:

*Interviewer: And I guess they will have given you instructions on the novel format. How did you find those, in terms of: were they clear, easy to follow, was there anything that you missed out or thought was explained well?*

*A8: Well, thinking back I think it was all quite clear. They did give a lot of detail about what was expected for the report and what to expect throughout the process. Um, so, yeah it was all fine.*

*Interviewer: Okay, and did you ask additional questions, or did you just get everything you needed from the instructions?*

*A8: I think we asked additional in terms of how much detail they wanted, whether it was to submit kind of like, almost like the grant application, really detailed proposal, or if they wanted just what we would write up for a manuscript, and they said they’d like to see a full proposal really, protocol.*

A8 stands out as an example of a good communication experience but we only identified this after specifically probing them on the topic. This reveals a potential asymmetry in our interviews. People told us extensively about the problems they had with communications, but partnerships clearly were successful in their communications because multiple submissions were received, reviewed, or granted IPA. This may be because failures in understanding and communications are more noticeable and memorable than successes or that we, as interviewers, did not ask specifically about successes. Still, even if communications were better than our interviews indicate, interviewees such as R5 had ideas that could help improve communications further. Specific ideas included providing templates for article or grant submissions, delivering information through figures, videos, and images instead of words, making information more eye-catching, and providing guides to frequently asked questions or common misconceptions.

### Influence on study design

Interviewees believed elements of the partnership processes helped to improve the designs of the submitted studies. A8, F3, E8, E5 and E4 even stated that this was one of their motivations for getting involved in partnerships. Interviewees’ testimonies indicated two mechanisms by which partnerships can affect study designs.

The first mechanism was the requirement to submit a detailed study plan before data collection. E8 argued that partnerships have the benefits of pre-registration, which they believed could improve research quality. A7 and A6 concurred with the opinions of E8; they both felt the need to justify their choices and provide a detailed methods section before data collection improved their respective studies.

*Interviewer: I was wondering what you meant by rigour, and what sort of particular things you think that the application process means that it forces rigour?*

*A6: Well, I think any time that you are forced to do a complete, detailed method section is one that – that’s what I mean by rigour, because I think it’s very easy for people when they’re writing grants to hand wave things that they are unsure of how they’ll actually accomplish, and have that be a problem for future-research-person, and current-applicant-person doesn’t need to worry about that because, if we get the grant it’s our problem them. But you can’t do that if you have to have your full methods there because you can’t just make things up.*

The second mechanism was peer review. Almost all reviewers, along with M2, E1, E2, E5, E8, F1, F4 and F6, asserted that peer review feedback at Stage 1 could improve the study design. A1, A2, A3, A6, and A8 supported this belief; they thought the feedback improved their study design and A4 found the feedback helpful. A1 and A8 liked how reviewers from the funder and editor gave different feedback.

*Interviewer: I was just wondering how you felt the review process went.*

*A1: Yeah. Okay, I see. Yeah, I think it was relatively light touch, to be fair, the review process, but it still is an additional review process, so it does add a bit of extra work. But I didn’t find it too problematic and I guess one of the positives, of course, is that you get additional information which also helps you improve your own study. So that’s a definite plus, because some of the reviewers picked up things that weren’t picked up by the reviewers of the grant application.*

More rounds of review with the same or different reviewers could mean more or better feedback to help authors improve their studies. However, A7 exemplified the risks associated with more peer review. They had a negative review experience, calling it the “longest, most difficult [revise and resubmit] process I’ve ever done in my life”. The main reason for this was a malignant reviewer. They stated:

*A7: With a typical [review and resubmit] you have your set of reviews, you send in revisions, and then you might have those revisions sent back to the original reviewers, or some journals the editors just make the decision. But you almost never in political science have more than two sets of R&Rs. This process, I’ve lost count. I think we’ve gone back and forth with the reviewers maybe four times at this point. Even after, okay, they’ve accepted the registered report, we’re free to collect data, we collect the data, we write it up and then the set of reviews they sent us this spring, one of the reviewers was like two paragraphs on how much he still hates our theory and doesn’t understand it and ‘you seem to be clinging to the [X] hypothesis, I just don’t understand this,’ and then bringing in all this literature he thought we should have incorporated and it’s like, no! We time stamped the registered report, we can’t go back and change the theory – what are you doing? Then the editor was like, if you could make these changes at the reviewer’s request then we can accept. It’s like, what? I mean, it’s just wild.*

A7’s experience chimes with A6, who speculated that more rounds of review increase the chances of bad reviewers. Only authors mentioned this risk, possibly because they are the target of the reviews so are more likely to experience the risks of reviewing. A7 proposed “strong editors” as a solution to malignant and unhelpful reviewers:

*A7: I just feel like this process can give an outsized role to dick reviewers and if editors don’t reign them in or change them this could be… So yeah, definitely there were times when I thought about withdrawing because I was like, we’re never going to satisfy this person, they fundamentally disagree with what we’re trying to do.*

We inferred from A7’s description that they would have liked the editor to have overridden some of the reviewer’s suggestions and advised the authors not to accept them. Our interviewees identified multiple ways in which partnerships can influence study design. This suggests that how partnerships decide to structure their peer review processes, such as the steps they take to mitigate risks of unhelpful reviewers, may have major impacts on the research they fund and publish.

### Appropriateness of partners

All editors, matchmakers, and funders had a positive relationship with their external partners. Of those who explained why, the most common reason the partners worked well together was because their ultimate goals or objectives aligned. These goals varied across partnerships, but interviewees believed the partners within them should work towards the same thing. Otherwise, disagreements and confusion could weaken the partners’ ability to work together.

*E2: I think the relationships with the funders have worked very well, they’ve been incredibly open and collaborative. I have to say I found it inspiring to see that there was so much alignment in terms of supporting reproducibility and openness from them, because it’s so important for us, but it’s always nice to work with somebody who has overlapping goals.*

*F5 I think it always comes down to, it’s really critical for funders to know that they’re trying to do. If I could put myself in the place of a grantee or a partner, I can’t think of anything more frustrating than a funder, even by accident, playing an elaborate game of find me a rock, no not that one!*

While funders, editors, and matchmakers valued the alignment of goals, all of them also needed to choose a partner that worked in the same discipline so that partners could fund and publish the same work. This is important because grant applicants would not want to submit to a partnership if they believed the partner journal was an inappropriate outlet for their work’s discipline. E2 and F5 stated that this “natural pairing” of disciplines informed their choice of partners. F4 described how this could create a dilemma for funders in partnerships:

*F4: Also, if you do it on a larger scale, for a funder like Cancer Research UK where they have a lot of different schemes, again the choice of the journals, you may have journals but you may not have people who are interested in publishing in those journals because of visibility, for instance. They might say, “It’s not one of the things I publish in”. Because you want to cover different disciplines, it might be more difficult to find the venue unless you go for a very generic one like PLOS perhaps, or PLOS Medicine, you might have all of the PLOS journals, but choosing one publisher only may not be the best solution, because the funders probably – I don’t know, but I would say – they might not want to be associated with one particular publisher, so I think logistic wise and the amount of workload and workflows, and then possibility the choice of the journals might be more difficult if you have to cover a lot of disciplines. For a niche area, it’s very clear they do publish already there, you know your researchers publish already in a venue, you may want to do that.*

A partnership with one journal may be easier to set up for niche research areas where a single particular journal publishes much of the research in that field but harder if the funder wants to cater to a range of disciplines. F4 proposed the solution of partnering with a “generic” journal but M2 suggests that this may not be enough to ensure the partner journal attracts applicants.

*M2: We know, there’s also of course the question of who’s the appropriate journal partner, which journal outlet would incentivise and interest this community the most. You know a lot of them aren’t too interested in a PLOS One journal article.*

Yet, researchers do not choose their publication outlet solely based on whether it matches their work’s discipline. For example, some authors expressed concern about the impact factor of the partner journal. IPA may reduce the risk of researchers being rejected based on their results and then resubmitting to another journal. However, A6 noted that the need to publish in high impact journals means IPA carries a potential risk.

*A6: The research metrics are how we survive in science … So, if I’m under-publishing, if I could be publishing in higher impact journals and I’m publishing medium or lower impact journals, then I’m not taking the most of my opportunities to hit those higher metrics.*

Authors, editors and funders agreed that if researchers believed they could publish in a higher impact journal they may not submit to the partnership, or may withdraw an accepted manuscript and instead submit it to a higher impact journal. A1 posited that this would worsen publication bias:

*A1: if you have a study that shows some positive effects that might be eligible for quite a high-impact journal, my concern would be that authors would actually forgo the opportunity to submit the paper to the journal that offers registered reports and therefore, essentially, it becomes a place where people submit non-significant, null findings, you see, because they wouldn’t get published in higher-impact journals.*

In line with A1’s concerns, F1 stated that two authors intended to withdraw because Plos One’s impact factor was too low. Funders and journals could place penalties on authors who withdraw but none did. F7 indicated why, arguing that penalties would undermine researchers’ freedom to choose their publication outlet. Our interviewees suggested that the partner journal’s impact factor may affect how many submissions the partnership can attract and retain. One way to make a partnership more desirable and minimise withdrawals would be to partner with a high-impact journal. A different solution suggested was consortia models, as we explain below.

***Consortia.*** Interviewees suggested various hypothetical alternative models for partnerships between funders and journals, beyond the existing partnerships between a single funder and a single journal. These included: a “marketplace” where journals “bid” for funded projects, a partnership between multiple funders and one journal or vice versa, and a partnership between multiple funders and multiple journals. As all of these models involve multiple funders or journals, we termed them “consortia” partnership models.

F1 was the first person we interviewed, and they suggested a consortia model as a way to make partnerships more attractive to researchers:

*F1: I would like to try to find a solution to give authors or awardees the opportunity to choose a difference place to publish their registered reports. This is one thing that I think will increase the opportunity for them to say “okay”. Because I don’t think they don’t like the style, the article type, it’s not against the article type, it is just the question of putting this article type in a good level journal that will satisfy their need for high level publication.*

We asked subsequent interviewees about their opinions on F1’s idea and E2 brought up the idea of “consortia” independently. Editors and funders suggested that consortia could bring several benefits to researchers, funders, and journals. They could protect researchers’ freedom to choose their publication outlet and make it easier to scale up partnerships to researchers from more disciplines if consortia included journals from a range of disciplines. They could also help to standardise partnership processes across multiple funders and journals, as suggested by E2:

*E2: I mean I think that the ideal scenario from my perspective for a journal like PLOS One, would be potentially to have some kind of agreement with a group of funders at one go, so a consortia type agreement, rather than having to do this on a per-funder-basis, where then you have to account for their individual funder processes, and how they want to do this, and the framework is different every time, which again means that you have to adjust your process every time and communicate to the authors differently every time.*

We cannot investigate consortia because none yet exist but our discussions with interviewees about consortia underlined the possible limitations of partnerships between one funder and one journal.

### Potential to reduce publication bias

In-principle acceptance (IPA) holds a key position in the scholarly discussion about RRs. As outlined in the Introduction, many editorials and commentaries about RRs argue that IPA will benefit research stakeholders. For one, it ensures the publication of negative or non-confirmatory results, reducing publication bias. Another benefit is that it provides authors with more certainty of publication before they collect data. KD investigated whether interviewees’ beliefs and expectations of the partnerships supported this conventional wisdom about IPA.

All interviewees either endorsed the benefits of IPA or had no major criticisms or concerns about it. Matchmakers, funders, and journals expected IPA to reduce publication bias, and, for some, it was one reason they created their partnerships. Funders were particularly positive about IPA, more so than editors. IPA had obvious benefits for funders since publication bias can result in funded research not being disseminated and researchers only submitting grant applications for projects likely to yield favourable results. F7 said they set up a partnership specifically because they wanted to incentivise “risky” research.

*F7: We wanted people, academic groups particularly, to come up with their kind of riskiest ideas; you know, if they had all the money in the world what would they choose to do with it? What would they think was a really good, interesting but perhaps risky Huntington’s disease project that would get us to therapeutics faster but, possibly might have a low chance of working? So obviously researchers, academics, in particular, become very sensitive and worried about not being able to publish stuff if it doesn’t work, so it brings in a whole mindset in science that only comfortable things are done. We wanted people to push the envelope, to use a terrible phrase, and think about doing some really risky science with the proviso that if it didn’t work, it wasn’t such a good idea in the end, as long as it was done properly it would still be published. So that’s what Registered Reports really do for us.*

Authors liked having IPA but gave little supportive evidence for IPA incentivising risky research. Five authors all agreed that IPA would reduce publication bias but only A5 and A7 commented on IPA in depth. A5 and A7 did not focus on the benefits of IPA resulting in a less biased literature. Instead, they focused on how IPA reduced the risk of getting rejected and needing to resubmit to another journal.

*KD: you’ve been talking about the benefits of like a pre-approved publication, can you just walk me through a little bit what you see the benefits of that being?*

*A5: Well it can be really painful picking a journal, and then submitting it, and then going through the process, and then whether they reject it or not, then you’ve got to find another journal, and then- often some, like if you haven’t published in a certain journal before, you don’t know how long they take, and some of them can be so painful, like they can take months before they even, you know, look at your work, or even make a decision, and then you’re just wasting time, like. It only takes a few months or so and then another group’s research comes out before yours, or, you know what I mean, I don’t know, and I’m an impatient person, I just wanted to have an answer and see if it gets through or not. So I don’t have to go through that at all because I know, you know, it’s pre-approved. I don’t have to worry about looking for a journal, I don’t have to worry about going to review and seeing if they’re going to accept it, minor changes, or what not.*

A7 also noted how, while IPA would reduce publication bias based on studies’ results, the fact that reviewers could have a greater influence on the study designs under an IPA system may actually increase publication bias against certain research questions and methods, at an earlier stage in the process.

*A7: I love the ability to not file drawer null results, I think open science is a really important move forward in every discipline, but I think that is the number one drawback, that reviewers kind of have a proportionally larger role in shaping the questions and measures that you want to use, and so for a younger researcher or for a researcher who’s trying to push the envelope on things like gender or how we understand racial and ethnic differences, or asking just questions outside of the box, I fear a little bit that those questions will be narrowed because reviewers are going to want to stick with the status quo or protect their paradigm or whatever it is that they’re wanting to do, and they’re going to keep you from measuring what you want to measure.*

While A5 and A7 provided useful insights into the benefits of IPA they were the exception. Compared to editors, matchmakers, and funders, most authors said little about IPA.

### Impact on reviewer workload

The RR format includes additional rounds of review and allows reviewers to influence the study design. It is uncertain whether this alternative peer review process increases the workload for reviewers. Despite some of our editors and funders worrying about additional reviewer time, only one reviewer, R5, felt they put in more work or time than normal. However, it is worth noting that most reviewers had not reviewed all stages of a manuscript so their opinions may change.

Two reviewers did not know that the studies they were reviewing had not begun data collection. One speculated that undestanding the format would have made them more enthusiastic to return. The other speculated that they would have pushed for more changes to the study design. Neither said they would have invested more work.

One explanation for why partnerships do not increase reviewer workloads may be how researchers allocate time for reviewing. A partnership publication could involve at least double the amount of review as a traditional publication because it has two rounds of review. This could increase reviewers’ workload for a single paper but may not affect workloads overall if researchers have a certain amount of time they commit to reviewing.

*R2: I wouldn’t be put off doing it because it’s a Registered Report, even though I know that sort of commits me to reviewing a second paper in some future time. Again, it would just count on my peer review quota, if you like, I wouldn’t see it as, that’s an additional paper than I would have had to have done anyway, it would just come out of the same quota so it wouldn’t matter.*

In contrast to the other reviewers, R5 said they put in double the time than normal because they felt more responsible for the study’s quality given that it had funding and that they could influence it.

*R5: I made a lot of comments and to some extent I felt at some point that I’m more a co-investigator, or you know co-designer of the final study than just a reviewer of someone else’s work. And so I had definitely given comments which would be going beyond the traditional review process, just because I felt maybe could make a difference.*

They distinguished their “co-designer” role from a normal reviewer role in that it involved more work and more complex work.

*R5: I had to think through the whole arch of the study. So actually this was doing a very, to some extent quite advanced design consulting. Normally this would be a kind of really thinking, you know, some of my comments were, “if you collect this kind of data, think for how you’re going to actually put it in the final table, in this journal”. Because actually I just remembered I also had to keep in mind the journal it’s going to go into and how much word count there will be. So it was actually, it was like planning for a study that would need to be publishing in a specific format, in a specific journal. Which is way more than normally you would do. So actually it was a lot of checking about, yeah, so way more things to consider than normally you would.*

R5’s perspective holds different implications for partnerships than those of our other interviewees. If partnership reviewers do more work and harder work, they may need more incentives, such as co-authorship or acknowledgement. However, most reviewers did not find reviewing for the partnerships to be more work than traditional reviewers and were willing to return for the review of the Stage 2 manuscript.

### Insufficient evidence

Funders, editors, and matchmakers were generally positive about their partnerships. None had abandoned the project because of difficulties, all had successfully established, or were establishing, a partnership, and all those who had put out a call for submissions had successfully received applications. However, all partnership handled very few submissions and only one had a completed publication when we interviewed them. The small number of submissions did not perturb funders, editors, or matchmakers because they considered the partnerships as pilots.

*M1: for what stage one was, it was just a proof concept, so it didn’t really matter how many submissions we got, it was just to prove that we could work together as a journal and a funder, to engage researchers to do a certain thing, publish a certain thing, and that happened, that was proved that we could do that.*

Partnerships are rare, young, and have scant literature on their impacts, implementation, or process. Piloting helped funders and journals minimise costs if they stopped the partnership, test out workflows, identify problems, improve later cycles, and gain experience with the process. Given that the pilot programmes had relatively low throughput, interviewees felt the partnerships were a successful proof of concept but were uncertain of their impacts.

Funders, editors, and matchmakers were hesitant to comment on certain issues. They demurred because the partnership was too young, received too few submissions, had too few completed publications, or had not been evaluated yet.

This lack of evidence led them to uncertainty about several issues, including: what needed improving (E1, E5, E8, F3, F5), the quality of submissions (E2, F2, F3), authors’ experience (F2, M1), reaction of their researcher community and potential authors (F5, M1), and appropriateness of journal (M2). To answer some of these questions some stakeholders conducted, or wanted to conduct, evaluations of their partnerships, such as interviews or surveys of authors and potential applicants. In contrast, authors and reviewers rarely struggled to answer questions because of lack of available evidence, though they sometimes could not remember specific details.

## Discussion

We conducted interviews with 32 authors, reviewers, journal editors, funders, and matchmakers across 6 partnerships between funders and journals offering Registered Reports. We interviewed each stakeholder at a point when their respective partnership was either receiving applications or being established, but only one partnership had published a RR. This means that most stakeholders had funded, authored, handled, or reviewed Stage 1 manuscripts but had not seen a RR through to publication.

This thematic analysis investigated the research question: “What are the experiences of the stakeholders (authors, reviewers, journal editors, funders) in the various partnership models?”. KD analysed the interviews using thematic analysis and constructed six themes that cut across all stakeholder groups.

Overall, interviewees were generally neutral or positive about their experience. None were overwhelmingly negative about the process or indicated that they would not participate again. Beyond this, KD found little consensus for any aspect of the partnership process that everyone liked or did not like. No one part of the partnership, such as IPA, is a universal benefit or cost for all stakeholders.

### Implications

Our study offers feedback that we hope will help organisations improve existing and future partnerships. Firstly, the Importance of communication with authors and reviewers theme indicates the importance of effective communication between all stakeholders. Our study found that some stakeholders did not understand the partnership process or what was required of them, revealing a risk that the implementation of the partnership differed from what funders, editors, and matchmakers planned. If partners want to implement their scheme as planned and avoid the unnecessary work of trying to correct any deviations, they need to know what misunderstandings occur, why, and how to minimise them.

We received much positive feedback about the partnerships. This was encouraging for the concept of partnerships. It may encourage organisations to set up new partnerships and for existing partnerships to continue or scale up. Partners agreed that they had good relationships, reviewers were willing to return and had similar workloads to conventional reviewing, and stakeholders believed the partnership improved study designs. The belief that the reviewer feedback or partnership workflow improved study designs is particularly encouraging. Given the frequent misunderstandings within our sample, it is likely that some reviewers in each partnership did not understand the process, but this may matter less if the workflow required to submit to a partnership can encourage authors to be more rigorous.

Taken together, our analysis indicates the potential for the sustainability and scalability of the partnership model in general. We identified factors that may facilitate or challenge partnerships in continuing or scaling up. Attracting and retaining authors was one factor. Future partnerships that want more applications may look to attract researchers from a wider range of disciplines but our “Appropriateness of partners” theme suggests authors will not apply if the partner journal is not relevant to their discipline. This could mean partnerships, specifically those between one funder and one journal, are only feasible with journals that cover a wide range of disciplines, or for niche research areas where the range of appropriate journals is limited.

One finding that we did not expect was how few authors spoke about IPA. We considered this surprising because IPA is a major distinguishing feature of RR publishing compared to traditional publishing and one that proponents of RRs expect to have major benefits for authors. Authors’ relative reticence on this topic may be because only one author (A8) had finished the final submission process, or because our questions encouraged them to focus on other topics, or because the benefits of IPA were not salient to them. Nevertheless, this gap in our author interviews means it is unclear whether the partnerships will realise the theorised benefits of IPA for authors.

Funders and editors also discussed other issues that eased or frustrated the set-up, management, and future of their partnership. These issues were only mentioned briefly or not at all in the themes because only funders and editors discussed them. They are mentioned here because future partnerships may want to consider them. Maintaining independent editorial and funding decisions was important to funders and editors, and they believed they did maintain independence. Funders and editors found setting up the partnership took considerable work and time, mainly because setting up any new workflow and relationship had bureaucratic costs, such as agreeing legal contracts between partners. Funders and editors frequently established a manual approach to handling submissions, using emails to communicate with people instead of online management software. Interviewees were unhappy with a manual workflow. Management software may help this issue, but several interviewees found existing management software to be unfit for handling partnership submissions. Highlighting these design considerations and issues for future partnerships should help organisations plan and manage them.

### Strengths and limitations

Aspects of our study strengthen our confidence in our findings. We had excellent coverage across the different stakeholder groups, only failing to represent reviewers from the APLS-PLS partnership, see Table 1. Our considerable number of interviewees also means we probably sampled a substantial percentage of the entire population of stakeholders involved in existing partnerships, given that this population is so small. This suggests our sample is likely to be representative of our target population and our findings relevant to them. The good representation from all stakeholder groups also allowed us to triangulate ideas. Seeing if and how a theme could be constructed across all groups and partnerships revealed nuances in the theme and whether the theme was common or general enough to warrant investigation. It also allowed ideas to be corroborated from multiple perspectives, as demonstrated by E5 and R2. E5 speculated that reviewers were not reading their emails which R2, a reviewer from that partnership, confirmed. The anticipated benefits of using semi-structured interviews to answer our research question were also met. For example, if we felt an interviewee was focusing heavily on what they liked about the partnership we could prompt them to talk about anything they did not like, and vice versa.

However, several factors limit our understanding of the implementation of the partnership process. A8 was the only author to have completed the entire RR process, and no other stakeholder had seen a submission through to publication. This is reflected in the “Insufficient evidence” theme and particularly restricts our understanding of the later stages of the partnership workflow, such as the Stage 2 review, publication, and the aftermath of publication.

Reviewers and authors did not feel limited by insufficient evidence, possibly because they were reflecting on their experience with a specific paper, instead of generalising about the entire partnership. They did sometimes have issues with their memory. R1, R2, and R3, all struggled to answer some questions because they could not remember much of their relevant experience. Interviewees were unwilling to answer certain questions because they felt they had too little evidence, could not remember, or did not realise that the study came from a partnership, as was the case with some reviewers. To help address these unanswered questions, future research could: follow up with authors or reviewers we interviewed once they complete Stage 2, examine more partnerships after they produce complete publications, or assess stakeholders’ experiences multiple times during partnership processes instead of after.

Despite these limitations and our narrow focus on partnerships, our interviews could provide a valuable perspective on the experience of RRs more broadly. Our interviews bring an alternative perspective to other sources because our authors and reviewers appear to come from a different audience than RR advocates. In our pilot interviews, we interviewed a self-selected sample of people who had been involved in RRs, most of whom believed that RRs or open science were beneficial. Maybe unsurprisingly, authors and reviewers were incredibly positive, sometimes passionate, about the benefits of RRs. In our partnership interviews, authors and reviewers were noticeably more ambivalent. Future researchers could examine existing data on RR experiences such as blog posts, journal articles, tweets, recordings of presentations and talks, and other social media posts, as well as our interviews. Such an analysis would provide more evidence for the impacts of RRs and maybe explain the discrepancy between our interviews and public reflections.

RC, JT, and KD had relatively little experience with interviews, thematic analysis, and qualitative research in general. Ideas like reflexivity, positionality statements, negative case analysis, thick description, prolonged engagement, and data saturation were unfamiliar to us. This was one reason as to why all three authors attended almost all interviews. We could support each other, provide a backup in case of internet issues, and provide feedback and possible follow up questions in real time. Alternating who was the interviewer meant we could learn from each other, gain a rich understanding of the interviews, and avoid fatigue. Having three simultaneous interviewers would be potentially intimidating in a face-to-face interview, but the virtual setting of the interview allowed the non-interviewers to turn off their video and microphone and listen without imposing on the interviewee.

The transparency of the paper was also restricted by a need to protect interviewees’ identity. We could not ensure the anonymity of data as rich as contained in the interviews, especially since it was essential for our analysis to link each interviewee with the partnership in which they participated. To protect interviewees’ identity whilst also sharing our data, we shared it as “Controlled data” on the University of Bristol’s data repository ^22^ which restricts access to bona fide researchers who will use the data for appropriate research purposes.

## Conclusion

Our thematic analysis of 32 semi-structured interviews produced six themes regarding the experiences of stakeholders involved in partnerships to fund and publish RRs: “Importance of communication with authors and reviewers”, “Influence on study design”, “Appropriateness of partners”, “Potential to reduce publication bias”, “Impact on reviewer workload”, and “Insufficient evidence”. The themes describe how partnerships between a funder and RR journal work in practice, their benefits, and potential pitfalls. Readers who apply to, review, set-up, or implement such partnerships should find our analysis helpful in developing their workflows and getting the most out of their experience. For example, our analysis provides insight into choosing a partner organisation and how to communicate with relevant stakeholders. Our analysis also provides insights into the feasibility of the continuation and expansion of partnerships. Our companion paper on the feasibility of a RCT of partnerships discusses this in greater detail ^72^.

## Data availability

### Underlying data

The study data are hosted on the University of Bristol’s online data repository ( data.bris) as controlled data at: https://doi.org/10.5523/bris.1m38wyz9gvzo52i2kpecr8w6kb.

It was essential for our analysis to link each interviewee with the partnership in which they participated and their role within it. Therefore, this stringent level of data control was chosen because some interviewees may be identifiable from their transcripts.

To access the data in data.bris, bona fide researchers will need to secure a Data Access Agreement from their host institution. With their host institution’s approval, a request for access will be judged by the repository’s Data Access Committee.

More information about Controlled Data access requests is available at: https://www.bristol.ac.uk/staff/researchers/data/accessing-research-data/.

### Extended data

It was difficult to share a list of codes used in the thematic analysis and coded transcripts because KD did not conduct all the thematic analysis in NVIVO. Much of the analysis was done manually on pieces of paper, word documents, and Excel spreadsheets. Instead, we shared as many coded segments of text as possible to provide a detailed example of the coding. This raised the question of “what” qualitative data should be shared. The iterative process of designing, refining, and analysing the interviews created a huge amount of data, including multiple versions of interview guides, coded transcripts, codebooks, NVIVO projects, and field notes. We shared the data we believed understandable and useful to others, but we struggled to find formal or informal guidelines on what qualitative data to share, so a larger conversation on the topic may be necessary.

Open Science Framework: Registered Reports funding partnerships: a feasibility study. https://doi.org/10.17605/OSF.IO/A7XS6 ^71^.

This project contains the following extended data:

- Protocol_RRFM_v1.0.pdf (the study protocol)
- coded-extracts-sample.csv (examples of codes and their relevant text.)
- interviewer-characteristics.csv (characteristics of the three interviewers, KD, JT, and RC, such as their credentials, occupation, gender, etc.)
- The 'Code' folder contains 4 files:
  - README-code.txt (instructions of how to use and understand the code)
  - 0-nvivo-export-options-anon.jpg (image to explain exporting from NVIVO)
  - 0-nvivo-export.txt (directions for exporting from NVIVO)
  - 1-collate-coded-text.R (code to collate files exported from NVIVO into a single csv file)
- The 'Efficiency Questionnaires' folder contains 2 files:
  - Efficiency_questionnaire_for_funder_CRUK_GRAND.pdf (the blank questionnaire sent to the funders at CRUK and GRAND, used to understand what data are accessible and shareable that could help to measure the efficiency of the funding-to-publication process.)
  - Efficiency_questionnaire_for_journal_PLOS.pdf (the blank questionnaire sent to editors at PLOS, used to understand what data are accessible and shareable that could help to measure the efficiency of the funding-to-publication process.)
- The 'Ethics' folder contains 3 files:
  - consent-form.pdf (the consent form used to obtain informed consent before the interview.)
  - debrief-sheet.pdf (the debriefing information given to participants after the interview.)
  - participant-information.pdf (the participant information document given to participants before the interview.)
- The 'Interview Guides' folder contains 5 files:
  - interview_guide.rmd (the R Markdown file used to knit the most recent interview guides. Different sets of questions are knitted by setting the params on lines 8–12 and choosing the appropriate stakeholder(s).)
  - interview_guide_authors.docx (the most recent version of an interview guide used when interviewing authors.)
  - interview_guide_editors.docx (the most recent version of an interview guide used when interviewing editors.)
  - interview_guide_funders.docx (the most recent version of an interview guide used when interviewing funders.)
  - interview_guide_reviewers.docx (the most recent version of an interview guide used when interviewing reviewers.)

Data are available under the terms of the Creative Commons Attribution 4.0 International license (CC-BY 4.0).
