# Supplementary material for: A qualitative analysis of stakeholder experiences with Registered Reports Funding Partnerships
Source: Wellcome Open Res. 2021 Sep 14;6:230. [Version 1] doi: 10.12688/wellcomeopenres.17029.1 (PMC8672223; doi:10.12688/wellcomeopenres.17029.1)
Supplement: Peer review history

# Peer review: Reviewer response for version 1

**DOI:** 10.21956/wellcomeopenres.18810.r47189

**Published:** 2021-12-14

**Authors:** Kyoshiro Sasaki

**Affiliations:** 1 Faculty of Informatics, Kansai University, Takatsuki, Japan

The present study identified the experiences of the stakeholders (authors, reviewers, journal editors, funders) in the RRFPs by a thematic analysis of semi-structured interviews. As a result, there were six important themes: “Importance of communication with authors and reviewers”, “Influence on study design”, “Appropriateness of partners”, “Potential to reduce publication bias”, “Impact on reviewer workload”, and “Insufficient evidence”.

 The present study adopted the high transparency practice and provides rich data (i.e., the detailed appendix). These would be highly beneficial for future developments of the RRs and RRFPs. There seems to be no fatal problem and thus I'm happy to approve the present paper.

 Just a comment 1: As mentioned in the "Influence on study design" of the Results section, we often encounter bad reviewers as A7. Of course, "strong editors" is one of the solutions. Perhaps, as another solution for this issue, the idea of "Author-driven publishing" (e.g., Stern & O'Shea, 2018; Patterson & Schekman, 2018), which is adopted in *eLife*, might be valid.

 Just a comment 2: One of the ways to reduce the risk of authors withdrawing the IPA protocol papers after observing the outcomes to submit to a higher impact factor journal might be that these protocol papers would never be linked to a specific journal. That is, review communities (e.g., PCI), not journals, mainly address the process of Stage 1.

 Again, thank you for providing the valuable opportunity to review the interesting study.

 References

 Patterson, M., & Schekman, R. (2018). Scientific publishing: A new twist on peer review. *eLife*, *7*, e36545.

 Stern, B.M., & O'Shea, E.K. (2018). Scientific publishing in the digital age. Accessed December 14, 2021.

Is the work clearly and accurately presented and does it cite the current literature?

Yes

If applicable, is the statistical analysis and its interpretation appropriate?

Yes

Are all the source data underlying the results available to ensure full reproducibility?

Yes

Is the study design appropriate and is the work technically sound?

Yes

Are the conclusions drawn adequately supported by the results?

Yes

Are sufficient details of methods and analysis provided to allow replication by others?

Yes

Reviewer Expertise:

Experimental psychology, Cognitive science

I confirm that I have read this submission and believe that I have an appropriate level of expertise to confirm that it is of an acceptable scientific standard.

## References

[1] : A new twist on peer review. *eLife* .2018;7: 10.7554/eLife.36545 10.7554/eLife.36545 PMC601906429944117

[2] : Scientific Publishing in the Digital Age. *ASAPbio* .2018; Reference source

# Peer review: Reviewer response for version 1

**DOI:** 10.21956/wellcomeopenres.18810.r47188

**Published:** 2021-12-13

**Authors:** Ingeborg Meijer

**Affiliations:** 1 Centre for Science and Technology Studies, Leiden University, Leiden, The Netherlands

Review of the Wellcome Open Research submission: A qualitative analysis of stakeholder experience with Registered Reports Funding partnerships. 

 This review addresses the following aspects: methodology, the topic of research/findings and the broader context. It was made without reading the first reviewers’ comment, in order to be ‘unbiased’.  

 The methodology section was very detailed, and well elaborated, covering the way the qualitative work and interview analysis has been carried out. It is hardly ever described in such detail, nor is the availability of data. In that sense this manuscript is an example of how open science should be done. Also, the interviewer characteristics are important elements that could be included much more often in interview-based publications. The broad research question that showed no preconceived ideas and the buildup of the story line based upon empirical material is much appreciated by this reviewer who has a science background and is now working in the more theoretically based social sciences. There are however a few aspects that deserve a bit more attention. 

 In the recruitment part of the methodology, there is a substantial number of ‘no replies’ (19), which means that almost half is non-responsive. Has a non-responder analysis been carried out? Furthermore, could you explain how editors can also be funders? And on that string more specifically, who represents a funder? Is it a researcher being a funders’ (programme) committee member judging proposals, or is it more like a research manager, having a background in research, or is it the chair of the scientific advisory board of a funder? This is especially relevant taking into account the match maker respondents. In the material-section, the interview guide refers to ‘impact’ on research quality’. How is that defined? Finally, in the respondents’ quotes, there is on some occasions a time indication and in others it is missing. Is that on purpose, and if so, what determines the choice? 

 The topic of research - registered reports and registered reports funder partnerships -addresses a very new, small and still very early days approach to increase the quality of open and transparent scientific research, which is laudable. However, when reading the first sentence of the abstract with the term Registered Report and the Registered report funding partnerships in the second sentence without knowing what it is (and most readers probably don’t), it is not very inviting to read further. It would have helped me to start in the introduction with explaining why it has been developed, by whom and in what context. This is probably self-evident when you are working with it, but for outsiders it raises immediate questions as: how international is this, is it covering all fields of science, how representative are the 300 journals that offer RR, how ‘big’ is this, and so forth. So, I first did some background search to understand the topic better. Later in the text, some of my initial questions, were implicitly answered, although there is no reference to the fact that the partnerships only consist of life science journals and funders. I noticed though that in the list of 300 more fields are covered. The reason to carry out this research is to prepare for a randomized control trial, a typical life science instrument, comparing other funding processes against RR, and I think that the results of this research show that a RCT at this point in time is far too early. Whilst the research has been carried out very diligently, the starting point hasn’t been helpful: the existing partnerships not only started recently, they also vary greatly and not surprisingly there is divergence in opinions from the respondents. Hence it is impossible to assign outcomes and themes to a particular structure or organization of partnerships. In fact, the first theme deducted from the interview results points to the most important theme, communication, signaling that authors and reviewers often did not understand the partnership and RR process, or even did not recognize it as something else. At the same time the editors and funders claimed to be successful because multiple submissions were received, albeit that no clarity was given on the total number of submissions and granted IPA’s. Adding to this asymmetry, there is the question of mission and goal alignment which was so clearly highlighted by the funders and editors. There was no information on the actual reason for the authors to go with this particular research question for this particular RR approach. Why were they doing it, what is their motivation, in this regard also the non-repliers could be relevant? Especially when pointing out that RR’s might be an outlet for publishing ‘null’ results (which is actually valuable) or that they would withdraw and go somewhere else. A limitation of the one-to-one funder-journal partnerships, is also that it pre-selects for monodisciplinarity while the current challenges require more multi and interdisciplinary research which is hard to fund anyway, which of course is something that the authors can’t help. Yet it is no surprise that various alternative models were suggested, such as the funder-journal consortia. Furthermore, the conclusion on the benefits of the IPA is to my mind not fully supported by the results: That IPA is either being endorsed or had no major criticism, is not what I read from A5 and A7, and the fact that most authors said little on the IPA. It leads me to conclude that in a next version of the paper, I would highlight the asymmetry a bit stronger, maybe connected to a section on ‘publication cultures’. 

 The broader scope of this paper refers to experiments initiated by the Center for Open Science to increase the quality of research by reducing publication bias and increasing the rigour of the study design, in order to make science more open, transparent and reproducible. This all complies very much with the rightful global policy aims for open science (see also the Unesco’s recommendations: https://www.unesco.org/en/natural-sciences/open-science) and in that sense every experiment that helps to do so should be supported (and ideally substantiated by evidence that it really does improve science). Yet, what this paper clearly shows is that the dominant publication cultures for authors and reviewers alike shines through all the themes: the authors and reviewers didn’t understand the RR process, didn't read specific instructions (as I haven’t done in this case, I’m sorry), didn’t pick up it up as being different, and instead judged the RR against their usual publications’ practices questioning whether it would help them in e.g. ‘the research metrics to survive in science’ giving all sorts of insights on how they live through that such as: which journal to pick, many rejections and changing journal, major revisions and the challenge to keep it your own paper, the need for high impact journals and the struggle to get your work published, while doing your academic duty in reviewing without spending too much time on it. This is standard practice for all of them, and one that I would personally would like to get rid of. Any experiment will have to battle with that practice, and whilst many researchers are positive on the goals of the RR as such in theory (as they are for many more experiments), in the meantime they have to keep on doing what is required of them. I would expect more reflections on this in the discussion section, and it would certainly affect the strength of the conclusion that ‘taken together the analysis indicates the potential for sustainability and scalability of the partnership model in general….’ Another interesting aspect that deserves more attention is the relation between funders and journals in the first place: the common denominator between them is that they rely on the academics for review in the system that they serve. The independence of the science system as such may come under threat if these links between academics serving the (funder or journal or both) system becomes even closer. A researcher can carry many hats! It would be wise to consider not only the benefits but also the potential perverse effects. And while I think it is extremely interesting that funders and journals are discussing and experimenting with these modes of operation, it would be good if they also open up and broaden out the review process (both for funding and journals) by bringing in non-academics to balance out implicit biases.  

 Actually, by reading on these practices I have definitively spent more time on reviewing it, in particular the writing of this summary. In summary I would recommend minor adjustments to better scope and position the small and early RR activities against the backdrop of traditional publication culture, and highlighting the asymmetry in responses a bit stronger. 

 Now that I have read the comments of reviewer 1, I endorse his comments on the fact that it is more likely that authors will withdraw in stage 1 than in stage 2, as they probably are incentivized by the certainty of having a publication over the uncertainty of several rounds into the unknown. However, this cannot be taken from the results as stage 2 was not readily represented. 

Is the work clearly and accurately presented and does it cite the current literature?

Yes

If applicable, is the statistical analysis and its interpretation appropriate?

Not applicable

Are all the source data underlying the results available to ensure full reproducibility?

Yes

Is the study design appropriate and is the work technically sound?

Yes

Are the conclusions drawn adequately supported by the results?

Yes

Are sufficient details of methods and analysis provided to allow replication by others?

Yes

Reviewer Expertise:

responsible research and innovation (RRI), societal impact of research, open science, rewards and recognition

I confirm that I have read this submission and believe that I have an appropriate level of expertise to confirm that it is of an acceptable scientific standard, however I have significant reservations, as outlined above.

# Peer review: Reviewer response for version 1

**DOI:** 10.21956/wellcomeopenres.18810.r45894

**Published:** 2021-10-04

**Authors:** Brian Nosek

**Affiliations:** 1 Center for Open Science, Charlottesville, VA, USA; 2 University of Virginia, Charlottesville, VA, USA

I appreciated the detailed description of methodology. There is a clear emphasis on transparency of approach, positionality, and the iterative nature of engaging with the interview data to arrive at the results and conclusions.

 The interviews identified meaningful and concrete challenges with communication about the RR process, particularly how editors’, reviewers’, and authors’ pre-existing experience with peer review in the standard format may provide a script leading them to misunderstand or ignore information about how the RR model is distinct. This challenge is particularly evident at stage 2 when editors and reviewers who do not understand the process begin to inject requests for revisions that directly counter the theoretical rationale for RRs such as rewriting the theoretical rationale based on the observed outcomes. Failure to communicate and educate all stakeholders in the RR process is a significant threat to effective implementation of the model and could undermine adoption and realization of the intended value of RRs.

 On the risk of authors withdrawing Stage 1 accepted submissions after observing the outcomes to submit to a higher impact factor journal (page 10), did the authors mentioned by F1 actually withdraw the paper(s)? This is an often-identified concern, but I am not yet aware of it having occurred in practice. The guarantee of publication following resubmission is a powerful incentive against the uncertainty of withdrawing the work and starting peer review from scratch elsewhere. That is not to say that this will never occur, just that I suspect it is more a concern in theory than in practice. Moreover, my observation is that it is a greater concern of authors prior to Stage 1 submission when they anticipate that any new research they do will be the next big breakthrough, and much less during preparation for Stage 2 submission when the results are never quite as beautiful as anticipated and the bird-in-hand of pre-acceptance is exerting its motivational pull. I do agree that the consortia model provides powerful messaging for authors who have such concerns prior to initial submission of Stage 1 proposals.

 Page 10: It is not correct that no consortia models have been attempted. The Election Pre-Acceptance Challenge was a consortia model with a group of Political Science journals. Also, relatedly, the Attitudes, Identities, and Individual Differences study released a small amount of data for exploration and preparation of Registered Reports that could be submitted to a consortia of journals. However, that one is not strictly a RRFP because there was no funder, the complementary incentive to publication was early access to a valuable dataset for authors that submitted a Registered Report about their research plan with the dataset.

 The authors’ mention as a limitation that almost all of these interviews occurred prior to completion of the RR through stage 2 report. I agree that this is the most significant limitation of this research, particularly given my perception that authors’ and reviewers’ beliefs about RRs evolve based on actual experience. For example, I would be very interested to know authors’ reactions to IPA after they have their results and are preparing Stage 2 submission and after final publication of their Stage 2 report. I suspect that there would be a substantially greater realization of the pleasure and relief of just reporting what happened to get the publication finalized versus the familiar stress of wondering if and how the observed results can be presented to meet yet unknown reviewer demands.

 There have been a few other RRFP’s: Laura and John Arnold Foundation and *Social Psychology* (culminated in a special issue of *Social Psychology* published in 2014), Laura and John Arnold Foundation and *Association for Psychological Science* (funding the first 5 or 6 Registered Replication Reports at *Perspectives on Psychological Science* and then *Advanced in Methods and Practices in Psychological Science*), and Laura and John Arnold Foundation and the Election Pre-Acceptance Challenge (a consortium of political science journals participated; https://osf.io/qtd5n/). Another forthcoming one is with Templeton World Charity Foundation and Association for the Scientific Studies of Consciousness (list of journal partners TBA). Except for the Election Pre-Acceptance Challenge, COS played an administrative role (matchmaker in the language of the paper) that ranged from managing process and financial awards (funder delegated decision making to COS) to managing both the financial awards and the journal editorial duties (i.e., journal and funder delegated decision making to COS for editor and funder roles respectively). The delegation of roles to third parties provides an opportunity to address some of the administrative challenges for funders especially and journals in some special cases.

 I particularly appreciated the detailed appendix describing the available data files supporting the reported research.

Is the work clearly and accurately presented and does it cite the current literature?

Yes

If applicable, is the statistical analysis and its interpretation appropriate?

Yes

Are all the source data underlying the results available to ensure full reproducibility?

Yes

Is the study design appropriate and is the work technically sound?

Yes

Are the conclusions drawn adequately supported by the results?

Yes

Are sufficient details of methods and analysis provided to allow replication by others?

Yes

Reviewer Expertise:

metascience, psychology

I confirm that I have read this submission and believe that I have an appropriate level of expertise to confirm that it is of an acceptable scientific standard.
